# Association between the diet gut microbiota index and survival in adult cancer survivors: Findings from the National Health and Nutrition Examination Survey (2007–2018)

**DOI:** 10.1097/MD.0000000000045870

**Published:** 2025-11-21

**Authors:** Rong Cong, Zhipeng Liu

**Affiliations:** a Emergency Department, The First Hospital of China Medical University, Shenyang, China.

**Keywords:** all-cause mortality, cancer survivors, DI-GM, NHANES

## Abstract

The gut microbiota (GM) is increasingly recognized as a key factor influencing cancer progression, treatment response, and long-term survival. Dietary patterns are major modulators of GM composition and may thus impact clinical outcomes in adult cancer survivors. However, the association between GM-related dietary indices and mortality in this population remains unclear. We analyzed data from 2711 adult cancer survivors (≥20 years, defined by self-reported physician diagnosis) in the National Health and Nutrition Examination Survey 2007–2018. The dietary index for gut microbiota (DI-GM) was calculated from two 24-hour dietary recalls (in person and telephone). Due to the study design, the timing of dietary data collection relative to cancer treatment status was not available. Cox proportional hazards models with appropriate weighting assessed the association between DI-GM (continuous and quartiles) and all-cause mortality, adjusting for key sociodemographic and clinical covariates. Mediation analysis assessed the role of systemic inflammation. Compared to the lowest quartile (Q1), the highest quartile (Q4) showed a 29% lower risk of all-cause mortality (hazard ratio = 0.71, 95% confidence interval: 0.57–0.88, *P* = .002). While a trend of decreasing mortality risk was observed across all quartiles, only Q4 reached statistical significance. Dose–response analysis indicated a nonlinear inverse relationship, with the protective effect plateauing at a DI-GM score of 6.78. Mediation analysis suggested that systemic inflammation, particularly assessed by the systemic inflammation response index, statistically but modestly mediated this relationship (average causal mediation effect = 0.994, 95% confidence interval: 0.988–0.998), confirming a partial mediation. Cancer-specific mortality was not assessed due to data limitations. A higher DI-GM score, reflecting a GM-friendly dietary pattern, was independently associated with lower all-cause mortality among US adult cancer survivors. These findings support the potential role of GM-friendly dietary patterns as a modifiable factor associated with improved long-term survival in US adult cancer survivors.

## 1. Introduction

In recent years, the gut microbiota (GM) has emerged as a critical modulator of cancer progression, treatment response, and long-term prognosis. This focus builds upon the established evidence that general dietary patterns and overall diet quality play an important role in improving cancer prognosis, given that diet is a major determinant of GM composition and function.^[1]^ An increasing body of evidence suggests that the composition and function of the intestinal microbiome play an essential role in regulating host immune response, metabolic homeostasis, and systemic inflammation – mechanisms intricately linked with cancer development and survivorship outcomes.^[2,3]^ For cancer survivors, optimizing modifiable lifestyle factors such as diet may hold promise for improving prognosis through microbiota-mediated pathways.

Diet stands out as one of the most powerful modulators of the GM, capable of rapidly altering both microbial diversity and metabolic output.^[4,5]^ Consistently, diets rich in fiber, fermented foods, and diverse plant-based compounds have been linked to increased microbial richness and the enhanced abundance of beneficial taxa. Key examples include species like *Faecalibacterium prausnitzii* and *Akkermansia muciniphila*, both recognized for their anti-inflammatory and immunomodulatory effects.^[6–8]^ Conversely, the typical Western-style dietary pattern, characterized by high consumption of red and processed meats, refined grains, and saturated fats, is frequently associated with microbial dysbiosis and the promotion of pro-inflammatory profiles.^[9,10]^

The dietary index for gut microbiota (DI-GM) is a novel, composite scoring system specifically developed to evaluate an individual’s diet quality in the context of its potential impact on GM composition and diversity.^[11]^ This index integrates 14 distinct dietary components, encompassing both beneficial items (e.g., fermented dairy, legumes, whole grains, fiber) and detrimental items (e.g., red meat, processed meat, high-fat intake), to generate a score ranging from 0 to 13. Previous epidemiological investigations have demonstrated that a higher DI-GM score correlates with a reduced risk of several adverse health outcomes, including depression, stroke, and metabolic dysfunction-associated fatty liver disease.^[12–14]^ Despite this accumulating evidence, the potential prognostic role of DI-GM in cancer survivors, who represent a rapidly expanding and particularly vulnerable population, remains largely unexplored.

Cancer survivors often face substantially elevated long-term mortality risks stemming from factors beyond recurrence, such as late effects of treatment toxicity and the prevalence of comorbid chronic conditions.^[15]^ Identifying specific, noninvasive nutritional strategies that primarily target the GM represents a feasible avenue for improving long-term health outcomes within this group. While preliminary evidence suggested that GM-modulating diets can both enhance the efficacy of immunotherapy and reduce systemic inflammation in patients with active cancer,^[16]^ no large-scale, population-based study has yet evaluated the association between the DI-GM and all-cause mortality among adult cancer survivors.

To address this knowledge gap, the present study sought to investigate the association between the DI-GM score and all-cause mortality in adult cancer survivors. We utilized data from the US National Health and Nutrition Examination Survey (NHANES) spanning 2007 to 2018, meticulously linked to mortality follow-up records through 2019. Our central hypothesis was that higher DI-GM scores would be associated with a lower risk of mortality in this specific patient population. Furthermore, we aimed to explore potential nonlinear dose–response relationships and assess whether established inflammation-related biomarkers might mediate the observed associations.

## 2. Materials and methods

### 2.1. Study population

**Figure 1.**
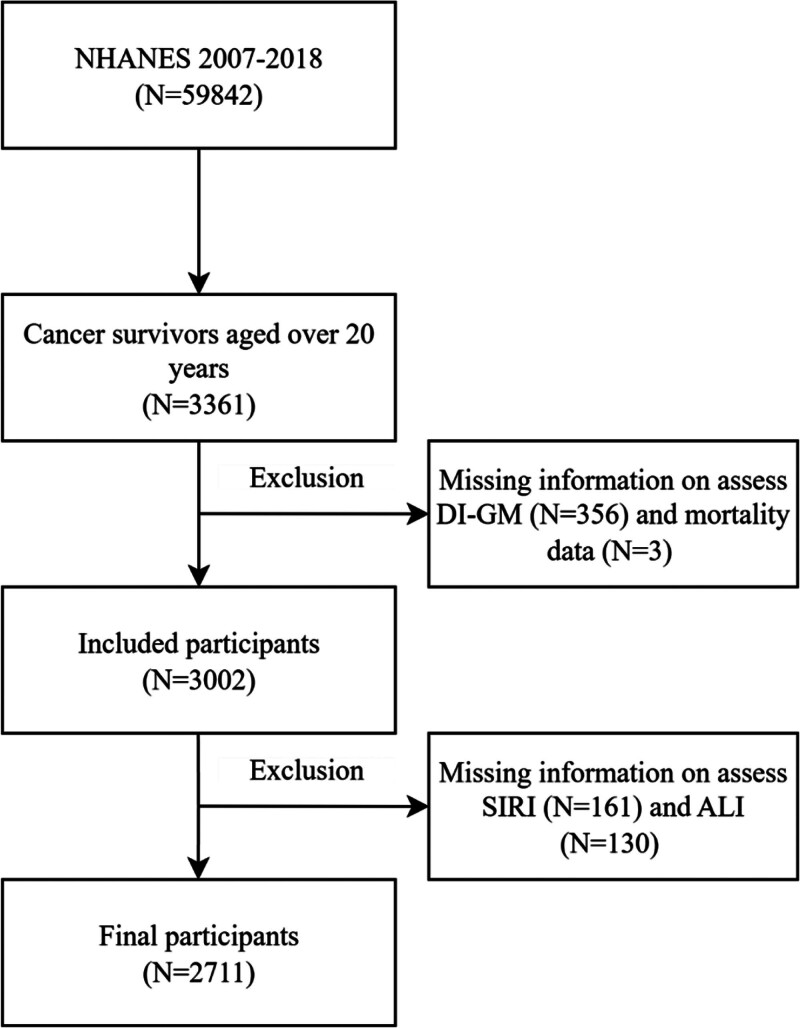
Flowchart for inclusion of study participants from NHANES. ALI = advanced lung inflammation index, DI-GM = dietary index for gut microbiota, NHANES = National Health and Nutrition Examination Survey, SIRI = systemic inflammation response index.

A total of 3361 cancer survivors aged ≥20 years were identified from the NHANES 2007–2018 survey cycles based on self-reported physician diagnoses. It is important to note that due to the cross-sectional design of NHANES, the specific stage of cancer, type of treatment received, or whether participants were disease-free or actively undergoing treatment at the time of the survey and dietary assessment is not available. The term “cancer survivors” in this study therefore refers to all adults with a history of cancer diagnosis. We first excluded participants with missing information necessary to calculate the DI-GM (N = 356), followed by those with missing mortality follow-up data (N = 3). We further excluded participants lacking data to assess the systemic inflammation response index (SIRI; N = 161) or the advanced lung cancer inflammation index (ALI; N = 130). After applying these exclusion criteria, the final analytic sample comprised 2711 participants with complete data on dietary intake, inflammation indices, mortality, and relevant covariates (Fig. 1). To assess potential selection bias from this complete case analysis, we compared the final analytic sample (N = 2711) with the excluded participants (N = 650) concerning key baseline characteristics (e.g., age, sex, body mass index [BMI]) and found no substantial systematic differences, suggesting the bias is likely minimal.

### 2.2. Assessment of DI-GM

The DI-GM was used as the primary exposure variable. This composite index was constructed based on up to-date evidence linking specific food components to gut microbial composition and diversity, utilizing two 24-hour dietary recalls (1 in person and 1 telephonic) as per NHANES protocol. The DI-GM comprises 14 dietary components – 10 beneficial (e.g., fermented dairy, legumes, whole grains, fiber, avocado, broccoli, green tea, etc) and 4 adverse (e.g., red meat, processed meat, refined grains, high-fat diet). Scoring was performed based on sex-specific median intake levels. A point was assigned for each beneficial component consumed above the median and for each adverse component consumed below the median (except for high-fat diet, which used a 40% energy threshold). The resulting DI-GM score ranged from 0 to 13, with higher scores indicating a GM-friendly dietary pattern.^[11]^

### 2.3. Outcome ascertainment

Mortality status and duration of follow-up were determined through linkage with the national death index using unique identifiers. The primary endpoint was all-cause mortality. Survival time was defined as the number of months from the NHANES interview date to the date of death or censoring (PERMTH_INT), whichever came first. Participants without mortality records were censored at the end of follow-up.

### 2.4. Definitions of mediating variables

We included the SIRI and the ALI as inflammatory mediators. These indices were calculated from laboratory data collected at the baseline NHANES examination (2007–2018). SIRI reflects systemic inflammatory status based on circulating immune cell counts, while ALI incorporates both inflammation and nutritional status indicators. Both have been associated with prognosis in cancer and chronic diseases.^[17,18]^

### 2.5. Covariates

We included several potential confounding variables based on previous literature,^[19,20]^ including age, sex, race, educational attainment, poverty income ratio (PIR), and BMI. Clinical conditions such as hypertension and diabetes status were also considered. These factors have been commonly associated with both inflammation and mortality risk in cancer survivors and were adjusted for in multivariable analyses to reduce potential confounding.

### 2.6. Statistical analysis

Cox proportional hazards regression was applied to evaluate the association between DI-GM and all-cause mortality, adjusting for potential confounders. Given the complex sampling design of NHANES, all analyses incorporated appropriate sampling weights, strata, and primary sampling units to generate nationally representative estimates, following the analytical guidelines provided by the National Center for Health Statistics. Mediation analysis was performed using bootstrap methods (1000 replications) to estimate the indirect effect mediated by SIRI and ALI. Subgroup analyses were conducted by stratifying participants based on demographic and clinical characteristics. Sensitivity analyses included excluding participants aged over 80 years and those with hypertension or diabetes to assess the robustness of the findings. All analyses were conducted using R software (version 4.5.1). We primarily utilized the survey package for incorporating the complex sampling design (weights, strata, and primary sampling units) of NHANES. The survival package was used for Cox proportional hazards regression. Restricted cubic spline models were fitted using the rms package, and mediation analysis was performed using the mediation package. A 2-sided *P* < .05 was considered statistically significant.

## 3. Results

### 3.1. Characteristics of the included participants

A total of 2711 cancer survivors were included in the final analysis. The baseline characteristics of participants stratified by DI-GM groups are presented in Table 1. The mean age of participants was 65.4 years, with a slightly higher proportion of females (52.3%) than males (47.7%). Participants with higher DI-GM scores tended to be older, predominantly non-Hispanic White, and more likely to have higher educational attainment and higher income-to-poverty ratios. Conversely, a lower proportion of participants with higher DI-GM scores were obese, hypertensive, or diabetic. Significant differences in demographic and clinical characteristics were observed across DI-GM groups, including age, gender distribution, race, educational level, PIR, BMI category, hypertension, and diabetes status (all *P* < .05).

**Table 1:** Baseline characteristics of participants by DI-GM group.

Character	Overall	DI-GM group				*P*-value *
		0–3	4	5	>5	
Participants (deaths)	2711 (621)	489 (150)	525 (135)	603 (120)	1094 (216)	
Age (SD)	65.37 (13.89)	65.25 (13.28)	64.36 (15.26)	64.75 (14.26)	66.25 (13.22)	.039
Gender (deaths, %)						.026
Male	1292 (374, 47.7)	257 (68, 52.6)	261 (85, 49.7)	284 (86, 47.1)	490 (135, 44.8)	
Female	1419 (240, 52.3)	232 (48, 47.4)	264 (47, 50.3)	319 (49, 52.9)	604 (96, 55.2)	
Race (deaths, %)						<.001
Hispanic	358 (38, 13.2)	73 (11, 14.9)	85 (8, 16.2)	74 (11, 12.3)	126 (8, 11.5)	
Non-Hispanic Black	365 (79, 13.5)	92 (21, 18.8)	87 (23, 16.6)	93 (18, 15.4)	93 (17, 8.5)	
Non-Hispanic White	1843 (478, 68.0)	298 (79, 60.9)	332 (96, 63.2)	402 (100, 66.7)	811 (203, 74.1)	
Other	145 (19, 5.3)	26 (5, 5.3)	21 (5, 4.0)	34 (5, 5.6)	64 (3, 5.9)	
Education (deaths, %)						<.001
Above High School	1554 (280, 57.3)	236 (46, 48.3)	266 (57, 50.7)	338 (57, 56.1)	714 (120, 65.3)	
High School	602 (150, 22.2)	126 (26, 25.8)	120 (26, 22.9)	146 (32, 24.3)	210 (66, 19.2)	
Less than High School	555 (184, 20.4)	127 (44, 26.0)	139 (49, 26.5)	119 (46, 19.6)	170 (45, 15.5)	
PIR (deaths, %)						<.001
<1.3	602 (156, 24.2)	136 (36, 30.2)	144 (46, 29.5)	138 (36, 25.0)	184 (38, 18.4)	
1.3–3.5	1011 (283, 40.6)	186 (58, 41.3)	201 (52, 41.2)	225 (65, 40.8)	399 (108, 39.8)	
>3.5	878 (138, 35.2)	128 (15, 28.4)	143 (26, 29.3)	188 (24, 34.1)	419 (73, 41.8)	
BMI (deaths, %)						<.001
<25 (underweight + normal)	721 (195, 26.6)	112 (35, 22.9)	106 (46, 20.2)	179 (41, 29.7)	324 (73, 29.6)	
25–29.9 (overweight)	950 (221, 35.0)	162 (38, 33.1)	188 (46, 35.8)	202 (50, 33.5)	398 (87, 36.4)	
≥30 (obese)	1040 (198, 38.4)	215 (43, 44.0)	231 (40, 44.0)	222 (44, 36.8)	372 (71, 34.0)	
Hypertension (deaths, %)						.004
Yes	1559 (431, 57.5)	292 (84, 59.7)	329 (98, 62.7)	351 (97, 58.2)	587 (152, 53.7)	
No	1146 (182, 42.4)	196 (32, 40.2)	193 (38, 37.0)	250 (38, 41.6)	507 (79, 46.3)	
Diabetes (deaths, %)						<.001
Yes	549 (162, 20.3)	120 (37, 24.5)	131 (35, 25.0)	120 (42, 19.9)	178 (48, 16.3)	
No	2064 (432, 79.0)	347 (75, 74.3)	383 (95, 74.5)	459 (87, 79.3)	875 (175, 83.1)	

BMI = body mass index, DI-GM = dietary index for gut microbiota, PIR = poverty income ratio, SD = standard deviation.

* *P*-value < .05 was considered significant.

### 3.2. Association between DI-GM and all-cause mortality risk

The association between the DI-GM and all-cause mortality was evaluated using Cox proportional hazards models (Table 2).

**Table 2:** Associations of DI-GM with cancer all-cause mortality.

Variables	Model 1		Model 2		Model 3	
	HR (95% CI)	*P* *	HR (95% CI)	*P* *	HR (95% CI)	*P* *
DI-GM	0.933 (0.893, 0.976)	.002	0.916 (0.876, 0.959)	<.001	0.945 (0.901, 0.991)	.021
DI-GM Levels						
0–3	1 [Reference]		1 [Reference]		1 [Reference]	
4	1.015 (0.791–1.302)	.910	1.015 (0.791–1.303)	.905	1.023 (0.790–1.325)	.862
5	0.885 (0.691–1.134)	.335	0.906 (0.707–1.162)	.438	0.918 (0.708–1.191)	.521
>5	0.758 (0.606–0.947)	.015	0.709 (0.566–0.887)	.003	0.811 (0.640–1.028)	.084

Model 1 was unadjusted.

Model 2 was adjusted for age, gender, and race.

Model 3 was adjusted for age, gender, race, education level, PIR, BMI, hypertension, and diabetes.

CI = confidence interval, DI-GM = dietary index for gut microbiota, HR = hazard ratio, PIR = poverty income ratio.

* *P*-value < .05 was considered significant.

When analyzed as a continuous variable, DI-GM was significantly inversely associated with mortality risk across all 3 models. In the unadjusted model (model 1), the hazard ratio (HR) for DI-GM was 0.933 (95% confidence interval [CI]: 0.893–0.976, *P* = .002). After adjusting for age, gender, and race (model 2), the association remained significant with an HR of 0.916 (95% CI: 0.876–0.959, *P* < .001). In the fully adjusted model (model 3), which additionally controlled for education level, PIR, BMI, hypertension, and diabetes, the HR was 0.945 (95% CI: 0.901–0.991, *P* = .021).

When categorized into 4 DI-GM groups with the lowest quartile (0–3) as the reference, the highest DI-GM group (>5) consistently showed a reduced risk of mortality in all models. Specifically, the HRs were 0.758 (95% CI: 0.606–0.947, *P* = .015) in model 1, 0.709 (95% CI: 0.566–0.887, *P* = .003) in model 2, and 0.811 (95% CI: 0.640–1.028, *P* = .084) in model 3. Intermediate DI-GM groups (4 and 5) did not demonstrate significant associations with mortality.

These findings suggested that higher DI-GM scores, reflecting a diet favorable to a healthy GM, may be associated with lower mortality risk among cancer survivors, highlighting the potential importance of gut microbiome-targeted dietary interventions in this population.

**Figure 2.**
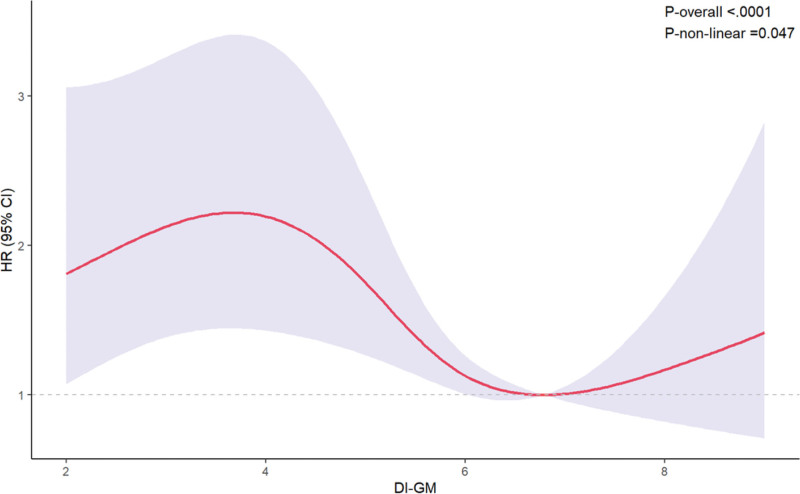
Association between DI-GM and all-cause mortality among cancer survivors using a restricted cubic spline model: the weighted Cox proportional hazards model with restricted cubic splines demonstrates a nonlinear inverse association between DI-GM and all-cause mortality risk. Shaded areas represent 95% confidence intervals. CI = confidence interval, DI-GM = dietary index for gut microbiota, HR = hazard ratio.

Using weighted Cox proportional hazards models with restricted cubic spline functions, we explored the dose–response relationship between DI-GM levels and all-cause mortality risk. The analysis revealed a significant association between DI-GM and mortality (*P*-overall < .0001). Moreover, the nonlinearity test indicated a statistically significant nonlinear relationship (*P*-nonlinear = .047). The spline curve suggested that mortality risk decreased with increasing DI-GM levels at lower ranges, reaching a plateau near the reference value (DI-GM = 6.78), after which the risk remained relatively stable. These findings suggested a potential dose–response association between DI-GM and mortality, characterized by a nonlinear protective effect (Fig. 2).

### 3.3. Subgroup analyses

As shown in Table S1, Supplemental Digital Content, https://links.lww.com/MD/Q741, the association between DI-GM and all-cause mortality was generally consistent across most subgroups. However, a stronger inverse association was observed among female participants (HR = 0.880, 95% CI: 0.821–0.943, *P* < .001), individuals with lower BMI (<25 kg/m²; HR = 0.854, 95% CI: 0.788–0.926, *P* < .001), and those with lower PIR (<1.3; HR = 0.898, 95% CI: 0.819–0.984, *P* = .022), as well as in participants without hypertension (HR = 0.911, 95% CI: 0.84–0.987, *P* = .022) or diabetes (HR = 0.922, 95% CI: 0.873–0.973, *P* = .003). Although no significant interaction was detected between DI-GM and most stratification variables (all *P* for interaction >.05), marginal interaction effects were observed for sex (*P* = .156), race/ethnicity (*P* = .212), and education level (*P* = .107), suggesting potential heterogeneity in these subgroups. These findings indicated that demographic and clinical factors may slightly modify the association between DI-GM and mortality risk, although the overall negative association remains robust across subgroups.

### 3.4. Sensitivity analysis

We conducted sensitivity analyses to evaluate the robustness of our findings. After excluding participants aged ≥80 years, the inverse association between DI-GM and all-cause mortality remained statistically significant (HR = 0.934, 95% CI: 0.877–0.995, *P* = .035). A similar association was observed after excluding individuals with self-reported hypertension and diabetes (HR = 0.941, 95% CI: 0.897–0.987, *P* = .012). In the subgroup analyses of DI-GM categories, participants in the highest DI-GM group (>5) consistently showed a trend toward lower mortality risk compared to those with DI-GM of 0 to 3, with borderline statistical significance across models. These results suggested that the observed association between DI-GM and mortality is generally robust and not entirely attributable to advanced age, hypertension, or diabetes (Tables S2 and S3, Supplemental Digital Content, https://links.lww.com/MD/Q741).

### 3.5. Mediation analysis

**Figure 3.**
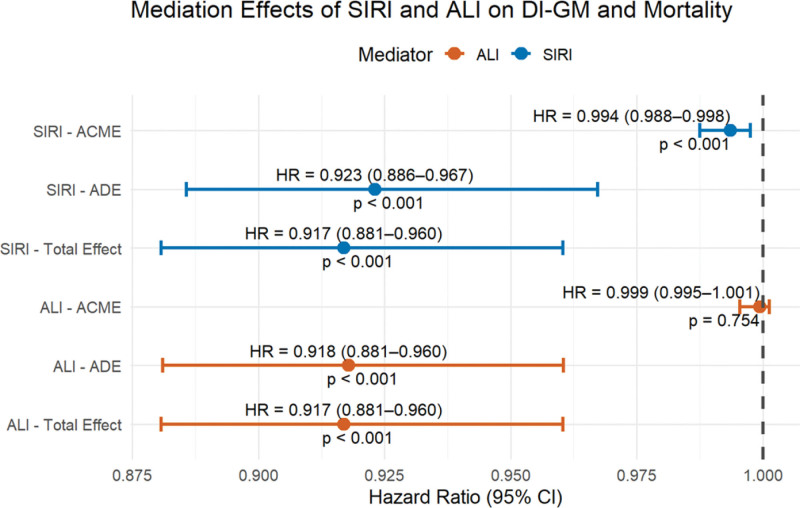
Mediation analysis of SIRI and ALI on DI-GM and mortality. ACME = average causal mediation effect, ADE = average direct effect, ALI = advanced lung inflammation index, CI = confidence interval, DI-GM = dietary index for gut microbiota, HR = hazard ratio, SIRI = systemic inflammation response index.

We further conducted mediation analyses to explore whether the association between DI-GM and all-cause mortality was mediated through systemic inflammation. As shown in Figure 3, the analysis using the SIRI indicated a significant indirect effect (average causal mediation effect, HR = 0.994, 95% CI: 0.988–0.998, *P* < .05), suggesting that SIRI partially mediated the inverse association between DI-GM and mortality risk. The direct effect of DI-GM remained significant (ADE, HR = 0.923, 95% CI: 0.886–0.967, *P* < .01).

Similarly, the mediation analysis with the ALI showed no significant indirect effect (average causal mediation effect, HR = 0.999, 95% CI: 0.995–1.001, *P* = .754), while the direct effect of DI-GM on mortality persisted (ADE, HR = 0.918, 95% CI: 0.881–0.960, *P* < .001). These findings suggested that systemic inflammation, particularly reflected by SIRI rather than ALI, may partially mediate the protective effect of DI-GM on all-cause mortality.

## 4. Discussion

In this nationally representative cohort of adult cancer survivors, we observed a significant inverse association between the DI-GM and all-cause mortality. Higher DI-GM scores, reflecting a diet more favorable to GM health, were consistently associated with lower mortality risk after adjusting for key demographic, socioeconomic, and clinical covariates. The dose–response analysis further confirmed this association, demonstrating a nonlinear protective effect of higher DI-GM levels. It is important to note that while the highest quartile (Q4) showed a significant protective association with mortality in models 1 and 2, this association was borderline nonsignificant in the fully adjusted model (model 3), highlighting the substantial influence of socioeconomic and clinical covariates.

Our findings align with existing reviews demonstrating that adherence to overall healthy dietary patterns, such as those rich in plant-based foods, is associated with improved survival and prognosis in various cancer survivor populations.^[21]^ The DI-GM, by focusing on GM-friendly components, provided a mechanistic lens through which these established dietary benefits may be operating. Our findings expanded on previous research linking diet, GM, and chronic disease outcomes. Prior studies have established that dietary patterns rich in fiber, fermented foods, and plant-based components promote gut microbial diversity and beneficial taxa, which can modulate immune function and systemic inflammation.^[22–25]^ Several observational studies have reported associations between higher DI-GM scores and reduced risk of depression, stroke, and metabolic liver diseases.^[12,26,27]^ However, to our knowledge, this is the first large-scale study to evaluate DI-GM in relation to mortality risk among cancer survivors.

The biological plausibility of our findings is supported by growing evidence on the GM’s role in modulating systemic inflammation and immune responses – factors critical for cancer prognosis.^[28–30]^ A healthy GM composition may reduce translocation of pro-inflammatory microbial products and enhance immune surveillance, potentially lowering mortality risk.^[31,32]^ Our mediation analysis further suggested that systemic inflammation, particularly measured by the SIRI, statistically but modestly mediated the relationship between DI-GM and mortality. This finding aligns with the concept of gut–immune–inflammation interplay influencing cancer survivorship outcomes.^[33,34]^

Subgroup analyses revealed consistent inverse associations across most demographic and clinical subgroups. While numerically stronger effects were observed in women, individuals with lower BMI, and those with lower income, the formal interaction tests for all subgroups did not reach statistical significance (all *P* for interaction >.05). Although interaction tests were not statistically significant, these patterns suggested that the protective effects of a GM-friendly diet may be particularly relevant for vulnerable subpopulations. Sensitivity analyses excluding older adults and those with hypertension or diabetes confirmed the robustness of our findings, indicating that the observed association is unlikely to be confounded by these common comorbidities.

The strengths of this study included the use of a large, nationally representative sample with standardized data collection, robust mortality follow-up, and comprehensive adjustment for potential confounders. Additionally, our incorporation of NHANES-recommended weighting enhanced the generalizability of findings to the broader US cancer survivor population. However, certain limitations should be acknowledged. First, the observational design precludes causal inference. Second, dietary intake was assessed via self-report, subject to recall bias and measurement error. Third, the DI-GM score, while evidence-based, is a composite index and may not capture all relevant dietary factors influencing the gut microbiome. Furthermore, we could not account for potential changes in diet following a cancer diagnosis or treatment, which may introduce measurement error. Fourth, our study relied on a complete case analysis. While we conducted a comparison and found no substantial systematic differences between the included and excluded participants concerning key baseline characteristics, we acknowledge the inherent risk of selection bias. Lastly, unmeasured confounders, such as smoking/alcohol intake, physical activity patterns, and medication use, as well as clinical factors like cancer stage, type, and specific treatment regimens (which are not available in the NHANES public data), may still influence the observed associations.

Despite these limitations, our study has important clinical and public health implications. Given the rising number of cancer survivors worldwide and their elevated risk of chronic diseases and mortality, identifying modifiable lifestyle factors like diet is crucial. Our findings suggested that promoting dietary patterns favorable to GM health may offer a feasible, non-pharmacological strategy to improve long-term outcomes in cancer survivors.

## 5. Conclusion

In conclusion, our study demonstrated a robust inverse association between the DI-GM and all-cause mortality among adult cancer survivors in the US population. These findings supported the potential role of GM-friendly dietary patterns as a modifiable factor associated with improved long-term survival in US adult cancer survivors. Future research, including interventional studies, is warranted to validate DI-GM as a predictive marker and to explore its integration into survivorship care plans. Dietary modulation of GM may be associated with a feasible, non-pharmacological strategy to improve long-term outcomes in cancer survivors.

## Author contributions

**Conceptualization:** Rong Cong, Zhipeng Liu.

**Data curation:** Rong Cong.

**Formal analysis:** Rong Cong.

**Validation:** Zhipeng Liu.

**Writing – original draft:** Rong Cong.

**Writing – review & editing:** Zhipeng Liu.

## Supplementary Material


