# Supplementary material for: Association between the diet gut microbiota index and survival in adult cancer survivors: Findings from the National Health and Nutrition Examination Survey (2007–2018)
Source: Medicine (Baltimore). 2025 Nov 21;104(47):e45870. doi: 10.1097/MD.0000000000045870 (PMC12643585; doi:10.1097/MD.0000000000045870)
Supplement: Supplementary file 1 [file medi-104-e45870-s001.docx]

**Supplementary Table 1** Subgroup Analysis of DI-GM and Mortality

| character | HR (95% CI) | P value | p for interaction |
| --- | --- | --- | --- |
| Gender |  |  | 0.156 |
| Male | 0.945 (0.891, 1.003) | 0.062 |  |
| Female | 0.880 (0.821, 0.943) | <0.001 |  |
| Race |  |  | 0.212 |
| Hispanic | 0.813 (0.656, 1.007) | 0.058 |  |
| Non-Hispanic Black | 0.878 (0.77, 1.001) | 0.051 |  |
| Non-Hispanic White | 0.937 (0.891, 0.985) | 0.011 |  |
| Other Race | 0.693 (0.548, 0.877) | 0.002 |  |
| Education level |  |  | 0.107 |
| Less than high school | 0.890 (0.817, 0.968) | 0.007 |  |
| High school | 0.994 (0.907, 1.089) | 0.890 |  |
| Above High School | 0.911 (0.852, 0.973) | 0.006 |  |
| PIR |  |  | 0.446 |
| <1.3 | 0.898 (0.819, 0.984) | 0.022 |  |
| 1.3∼3.5 | 0.937 (0.877, 1.001) | 0.055 |  |
| >3.5 | 0.97 (0.883, 1.066) | 0.530 |  |
| BMI |  |  | 0.297 |
| <25 | 0.854 (0.788, 0.926) | <0.001 |  |
| 25-29.9 | 0.905 (0.837, 0.978) | 0.012 |  |
| ≥30 | 0.955 (0.886, 1.03) | 0.234 |  |
| Hypertension |  |  | 0.845 |
| Yes | 0.92 (0.872, 0.971) | 0.003 |  |
| No | 0.911 (0.84, 0.987) | 0.022 |  |
| Diabetes |  |  | 0.925 |
| Yes | 0.915 (0.841, 0.997) | 0.041 |  |
| No | 0.922 (0.873, 0.973) | 0.003 |  |

Abbreviations: DI-GM, dietary index for gut microbiota; HR, hazard ratio; CI, confidence interval;

PIR, poverty income ratio; BMI, body mass index

*P-value <0.05 was considered significant.

**Supplementary Table 2** Sensitivity Analysis of DI-GM Excluding Participants Aged 80 and Above

| Variables | Model1 | | Model2 | | Model3 | |
| --- | --- | --- | --- | --- | --- | --- |
|  | HR (95%CI) | p | HR (95%CI) | p | HR (95%CI) | p |
| DI-GM | 0.898(0.847,0.953) | <0.001 | 0.888(0.837,0.941) | <0.001 | 0.934(0.877,0.995) | 0.035 |
| DI-GM Levels | | | | | | |
| 0-3 | 1[Reference] |  | 1[Reference] |  | 1[Reference] |  |
| 4 | 0.894 (0.650-1.230) | 0.490 | 0.940 (0.683–1.290) | 0.702 | 0.962 (0.690–1.340) | 0.820 |
| 5 | 0.743 (0.540–1.020) | 0.070 | 0.782(0.567–1.080) | 0.134 | 0.815 (0.583–1.140) | 0.231 |
| >5 | 0.616 (0.462–0.820) | <0.001 | 0.597 (0.447–0.799) | <0.001 | 0.746 (0.548–1.010) | 0.062 |

Abbreviations: DI-GM, dietary index for gut microbiota; HR, hazard ratio; CI, confidence interval;

Model 1 was unadjusted

Model 2 was adjusted for age, gender, and race

Model 3 was adjusted for age, gender, race, education level, PIR, BMI, hypertension, and diabetes

*P-value <0.05 was considered significant.

**Supplementary Table 3** Sensitivity Analysis of DI-GM Excluding Participants with Hypertension and Diabetes

| Variables | Model1 | | Model2 | | Model3 | |
| --- | --- | --- | --- | --- | --- | --- |
|  | HR (95%CI) | p | HR (95%CI) | p | HR (95%CI) | p |
| DI-GM | 0.933(0.893,0.976) | 0.002 | 0.916(0.876,0.959) | <0.001 | 0.941(0.897,0.987) | 0.012 |
| DI-GM Levels | | | | | | |
| 0-3 | 1[Reference] |  | 1[Reference] |  | 1[Reference] |  |
| 4 | 1.010(0.791-1.300) | 0.910 | 1.020 (0.791–1.300) | 0.905 | 1.010 (0.780–1.310) | 0.943 |
| 5 | 0.885 (0.691–1.130) | 0.335 | 0.906(0.707–1.160) | 0.438 | 0.907 (0.700–1.180) | 0.463 |
| >5 | 0.758 (0.606–0.947) | <0.015 | 0.709 (0.566–0.887) | 0.003 | 0.795 (0.628–1.010) | 0.057 |

Abbreviations: DI-GM, dietary index for gut microbiota; HR, hazard ratio; CI, confidence interval;

Model 1 was unadjusted

Model 2 was adjusted for age, gender, and race

Model 3 was adjusted for age, gender, race, education level, PIR, and BMI.

*P-value <0.05 was considered significant.
